# Evolutionary divergence in a non-genitalic sexual contact character in the beetle genus *Choleva*

**DOI:** 10.7717/peerj.21266

**Published:** 2026-06-10

**Authors:** Mike Groenhof, Henk van der Meulen, Alina von Thaden, Menno Schilthuizen

**Affiliations:** 1 Evolutionary Ecology Department, Naturalis Biodiversity Center, Leiden, Netherlands; 2 Institute of Biology Leiden, Leiden University, Leiden, Netherlands; 3 GELIFES Institute, University of Groningen, Groningen, Netherlands; 4 Conservation Genetics Group, Senckenberg Research Institute and Natural History Museum, Gelnhausen, Germany; 5 DZG e.V., German Zoological Society, München, Germany

**Keywords:** Leiodidae, Coleoptera, Staphylinoidea, Trochanter, Non-genitalic sexual contact characters, Character evolution, Molecular phylogenetics, Microcomputed tomography, Tempo of evolution, Leg morphology

## Abstract

It has been argued that non-genitalic contact characters display similar evolutionary patterns as genitalic characters. We studied the shape of the male metatrochanter in the beetle genus *Choleva* (Leiodidae). We used multi-locus molecular phylogenetics to reconstruct the phylogeny for 19 species-level taxa, and quantified their metatrochanter shape by applying spherical harmonics to microcomputed tomography scans. As a control, we did the same for the (undifferentiated) male mesotrochanter. Finally, we obtained maximum likelihood estimates for Pagel’s *κ* for 31 multiple aspects of shape to estimate the tempo of evolution. We found that, while the overall shape of the metatrochanters has evolved in a continuous fashion over time, specific aspects of the shape have evolved punctuationally with speciation events. Unexpectedly, in the undifferentiated mesotrochanter, we also found evidence for punctuated evolution, which may reflect developmental coupling of leg morphology for the middle and hind legs.

## Introduction

Genitalia are among the most complex and rapidly evolving structures in the animal kingdom and are generally highly divergent even between closely related and otherwise morphologically near-indistinguishable species (Eberhard, 1985; Arnqvist, 1998). Besides primary and secondary genitalia, other “non-genitalic contact structures” show a similar pattern of divergence (Eberhard, 1985; Eberhard, 2004; Eberhard, 2010). Sexual selection has been thought to play a key role in explaining this diversity (Eberhard, 1985; Eberhard, 1996; Arnqvist, 1998; Eberhard, 2004; Eberhard, 2010; Simmons, 2014). Different mechanisms of selection have been proposed, the four main ones being: the mechanical and/or sensory lock-and-key (Shapiro & Porter, 1989; Masly, 2012), sperm competition (Parker, 1970; Waage, 1979), sexually antagonistic coevolution (SAC; Parker, 1979; Alexander, Marshall & Cooley, 1997; Eberhard, 2004), and cryptic female choice (CFC; Eberhard, 1985; Eberhard, 1996; Eberhard, 2010). These mechanisms are not necessarily mutually exclusive and may act simultaneously in the same character system, although sperm competition is less likely to be involved in the non-genitalic contact structures. The mechanisms may also be viewed as parts of the same continuum where at one end female choice causes species isolation by generating strong stabilising selection, and at the other end female choice causes directional selection with a continuous pattern of divergence (Simmons, 2014). Moreover, the overall strength of selection on intromittent and non-intromittent structures may differ, with selection being weaker on non-intromittent genital structures and weaker still on non-genital non-intromittent structures (Rowe & Arnqvist, 2011).

CFC has been considered one of the most common sexual selection mechanisms to cause genital divergence (Eberhard, 1985; Eberhard, 1996; Eberhard, 2010). It is a form of postcopulatory selection in which the female influences male reproductive success based on stimuli received during copulation, called copulatory courtship (Eberhard, 1985; Eberhard, 1996; Eberhard, 2010). Presumed examples of copulatory courtship are widespread (Eberhard, 1994), especially in invertebrates like arachnids (Peretti & Eberhard, 2010; Cargnelutti, Calbacho-Rosa & Peretti, 2021) and insects (Briceño & Eberhard, 2009; Eberhard & Gelhaus, 2009; Wulff et al., 2015; Briceño et al., 2016; Wulff et al., 2017; Wulff, Schöneich & Lehmann, 2018; Eberhard & Lehmann, 2019). Both intromittent and non-intromittent structures can be involved in copulatory courtship. For example, several species of male bush crickets have specialised organs called titillators that stimulate females internally (Wulff et al., 2015; Wulff et al., 2017; Wulff, Schöneich & Lehmann, 2018), whereas external copulatory stimulation by stridulation has been found in, for example, crane flies (Eberhard & Gelhaus, 2009).

Non-genitalic contact structures can also arise and evolve due to the previously mentioned mechanisms. In damselflies, males grasp the females with their cerci at the female thoracic plates prior to mating and both structures have been shown to co-evolve in a pattern of punctuated equilibria (McPeek et al., 2008; McPeek, Shen & Farid, 2009). These non-genitalic contact structures are important for species recognition and they may have evolved under lock-and-key processes that reduce disadvantageous hybrid coupling (McPeek et al., 2008; McPeek, Shen & Farid, 2009). Alternatively, in sepsid flies, it has been proposed that the interspecifically highly variable clasping organs on the male front femurs have evolved under CFC as concluded by the absence of any obvious mechanic or sensory locks, and by the finding that manipulation of the male front legs did not alter the success of grasping the female (Eberhard, 2001; Eberhard, 2002; but see Ingram et al., 2008).

**Figure 1:**
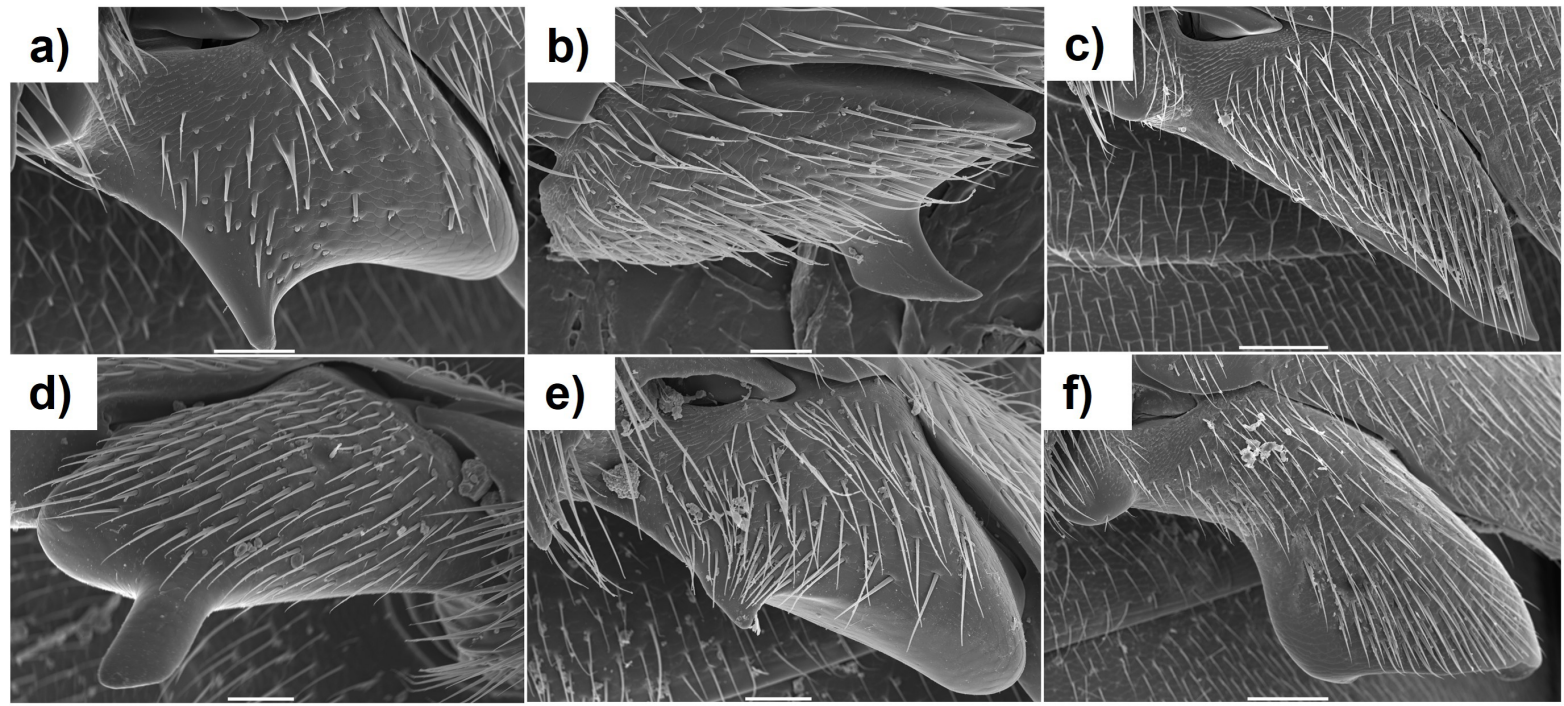
Scanning electron micrographs of male metatrochanters. Scanning electron micrographs of left (A–C, E–F) and right (D) male metatrochanters of six *Choleva* species, (A) *Ch.agilis*; (B) *Ch. angustata*; (C) *Ch. elongata*; (D) *Ch. glauca*; (E) *Ch. lederiana gracilenta*; (F) *Ch. oblonga oblonga*. Scale bars A, B, D, E, 50.

Members of the beetle genus *Choleva* (Leiodidae) are largely subterranean, have a Palearctic distribution and probably mostly feed on fungi and decaying animal matter (Krogerus, 1927; Van der Wiel, 1931; Benick, 1937; Schilthuizen, 1990). In this genus the metatrochanter, the segment at the basis of the hind femur, displays sexual dimorphism. While the female metatrochanter shows very little interspecific divergence, in the males, it is of complex, divergent, and species-specific shape (Fig. 1), although there is also intraspecific variability (Van der Wiel, 1931; Jeannel, 1936; Szymczakowski, 1963; Blas, 1980; Schilthuizen, 1990). Sex- and species-specific differences in setation and shape of the metatrochanter are also taxonomically informative in other insect groups, such as flower flies (Van Steenis et al., 2016; Mengual, 2018; Ramage, Charlat & Mengual, 2018), hemipterans (Xie & Liu, 2015), and beetles from the families Scarabaeidae and Chrysomelidae (Bezděk, 2020; Duarte & Grossi, 2020).

Images of male–female pairs involved in copulation suggest that during mating the male metatrochanter may come into contact with the distal end of the female elytra, the tergites of the last few abdominal segments, or even the genital tergite (Hubart, 1973; Thieren & Dethier, 2006). Given its great diversity in shape and size, the evolution of this non-genitalic contact structure may therefore, like those of genitalia, be driven by postcopulatory sexual selection by the female.

The manner in which selection drives macro-evolutionary divergence in a lineage may be gauged from analyses of the phylogenetic behaviour of a character. When the morphology of a structure is important for species recognition, as in the lock-and-key hypothesis, theory predicts that there should be strong stabilising selection on these traits and change should be rapid at speciation events to establish species boundaries (Shapiro & Porter, 1989; McPeek et al., 2008; McPeek, Shen & Farid, 2009; Masly, 2012; Stefanini et al., 2021). McPeek et al. (2008) and McPeek, Shen & Farid (2009) argued that traits subjected to such strong stabilising selection should thus show a pattern of punctuated character change over time, where characters change quickly during speciation events but change only slowly in between. In contrast, characters under sexual selection, either *via* CFC or SAC, should change in a more continuous fashion over time, as the processes driving divergence should be more or less independent of speciation events and characters are expected to evolve under the assumption of Brownian motion (McPeek et al., 2008; McPeek, Shen & Farid, 2009; Arbuthnott et al., 2010).

Pagel’s parameter κ can be used as a measure of the tempo of evolution (Pagel, 1994; Pagel, 1997; Pagel, 1999). If κ = 0 or κ <<1, this indicates that a character has probably evolved under a model of punctuated equilibrium, whereas if κ = 1, the branch lengths scale 1:1 with amount of divergence, which can be interpreted as a character that changes continuously over time (Pagel, 1994; Pagel, 1997; Pagel, 1999; McPeek et al., 2008; McPeek, Shen & Farid, 2009; Arbuthnott et al., 2010; Stefanini et al., 2021). In this study, we generate a new molecular phylogenetic reconstruction for a selection of *Choleva* species. Using μCT scanning, we also generate three-dimensional models for the male trochanter and, using Pagel’s κ (Pagel, 1994; Pagel, 1997; Pagel, 1999) infer the tempo by which its shape has evolved within the genus *Choleva*, based on metrics derived from the spherical harmonics framework (Shen & Mackedon, 2006; Shen, Farid & McPeek, 2009).

## Materials & Methods

### Specimens

Preserved specimens (both dry and in ethanol) were obtained from natural history collections. For the molecular work, only recently-collected, ethanol-preserved specimens were used; we obtained and used DNA sequences from 29 specimens of a total of 19 (sub)species and informal infraspecific taxa. For the morphological work, only male, dry-mounted specimens were used; in total, we obtained micro-CT scans for each of the same 19 ‘taxa’ that were included in the molecular phylogenetic reconstruction. In general, the intraspecific variability in male metatrochanter shape is very low compared with the interspecific variability (Jeannel, 1936). For this reason, and the fact that the 3D-morphological analysis is very time-consuming, we limited our morphological study to a single male per taxon. All samples and their details and metadata are provided in Table S1.

### DNA extraction

For each specimen, a single leg was crushed and homogenised using a micropestle and sterile sand. We used magnetic bead isolation (Ling et al., 2012), with beads produced following Rohland & Reich (2012). One hundred µL of Aljanabi & Martinez (1997) lysis buffer were added to the tissue sample, vortexed, and incubated overnight at 56 °C on a shaking incubator at 300 rpm. Afterwards, the samples were vortexed again to release residual DNA. Subsequently, 3.0x magnetic beads solution (300 µL) was added to the buffer and incubated for 10 min. The samples were then briefly vortexed and introduced into a magnetic rack, where they remained until the solution became completely transparent and all beads had been attracted to the magnet. Then, the supernatant was removed and 600 µL of 70% ethanol were added to wash the beads. This was achieved by incubating for 2 min and subsequently rotating the tube in the rack to move the beads along the side of the tube. Additionally, the washing buffer was gently pipetted up and down approximately 20 times after which it was removed and discarded. This step was repeated a second time with 300 µL of 70% ethanol. After removal, the pellet of beads was air-dried until very light crackling appeared (approximately 3 min). Finally, the DNA was eluted in 20 µL of RNase-free water. The beads were incubated in the elution buffer for 1 min and then again attracted to the magnetic rack, after which the supernatant containing the DNA was transferred to a new tube and stored at −20 °C.

### PCR amplification and sequencing

Six loci were amplified that have previously been used in phylogenetic studies in Cholevinae (Von Thaden, 2013; Schilthuizen et al., 2016; Fresneda, Grebennikov & Ribera, 2021). DNA samples were diluted to a concentration of approximately three ng/µL according to readings of an Implen Nanophotometer N60. One µL of diluted DNA sample was added to 24 µL of PCR master mix (200 µM dNTPs, 1X PCR buffer, 400 nM of forward and reverse primers and 1U of dreamTaq polymerase). The annealing temperature (Ta) of each primer pair was calculated using PerlPrimer v1.1.21 (Marshall, 2004) and are listed in Table S2 . Each primer was extended with the following tails to allow 96-well plate sequencing of a mix of several different fragments: (TGTAAAACGACGGCCAGT-{forward primer} and CAGGAAACAGCTATGAC-{reverse primer}). These tails do not affect the annealing temperatures. A standard PCR protocol was performed on a MiniOne PCR: Initial denaturation at 94 °C for 3 min, then 45 cycles of (i) denaturation at 94 °C for 30 s, (ii) annealing at Ta for 30 s, (iii) extension at 72 °C for 45 s per kb, then a final extension at 72 °C for 6 min. PCR products were sequenced in both directions by Baseclear B.V., Leiden.

### Phylogenetic analyses

The sequence data were loaded into Geneious Prime v2022.1.1. Forward and reverse sequences were aligned using Geneious alignment where the de novo assembly function was used to construct consensus sequences. In this way, most IUPAC ambiguity codes could be resolved, either automatically or manually. If needed, the automatic trimming was adjusted manually. In addition to these newly-generated data (all of which were uploaded to GenBank), several sequences from reliably identified specimens were taken from GenBank and from Von Thaden (2013). Data were aligned for each locus separately and primer regions were trimmed. To create a (sub)species-level dataset, multiple sequences for one locus were combined, with ambiguity codes for polymorphic sites. *Nargus algiricus* Portevin 1903 was added to serve as an outgroup (Von Thaden, 2013). All alignments were finally concatenated to form a total sequence length of 3,786 bp.

We then used jModelTest2 (Darriba et al., 2012; Guindon & Gascuel, 2003) to compute the best-fitting nucleotide substitution model for each locus. The number of substitution schemes was set to three and the Akaike information criterion with correction (AICc) was used to evaluate goodness-of-fit. This resulted in the following substitution models: GTR + I + Γ for CytB, nad1, COIa, and COIb, GTR + I for 28S, and SYM + I for 18S. MrBayes v3.2.7 x86_64 (Huelsenbeck & Ronquist, 2001) was used to construct a time-calibrated ultrametric Bayesian inference (BI) tree. A partitioned model was applied and the revmat, statefreq, shape, and pinvar parameters were unlinked for each locus. Average substitution rates in insects were found to be 0.58% per My in nuclear DNA (Andújar, Serrano & Gómez-Zurita, 2012) relative to 2.68% per My in mitochondrial DNA (Papadopoulou, Anastasiou & Vogler, 2010; Andújar, Serrano & Gómez-Zurita, 2012). These parameters were included in MrBayes, using a relaxed clock model. The analysis was then run for 5,000,000 Markov-Chain-Monte-Carlo (MCMC) generations with a sample frequency of 100 and a burn-in of 10,000 until the average standard deviation of split frequencies steadily fell below the 0.01 threshold. This also resulted in steady PSRF values of 1.000 to 1.002 and an average effective sample size above 100 for all parameters. A maximum-likelihood (ML) tree was constructed in RAxML (Kozlov et al., 2019), applying different substitution models to each locus, and rapid bootstrapping with automatic determination of sufficient bootstraps. We set the BI tree as a constraint to the topology.

### Micro computed tomography scanning

For each specimen, we obtained three-dimensional models for a meso and a metatrochanter, as follows. For dry, card-mounted specimens, pins and all labels were removed and the card with the glued specimen was inserted into a three mL plastic pipette tip to fix it in place during scanning. Micro-CT scanning was done with the Xradia 520 Versa 3D X-ray microscope (Carl Zeiss, Oberkochen, Germany), at 80.0 kV, 7.0 W, pixel size between 2.1–2.4 µm, rotation through 180° plus fanning, rotation step 0.11° per frame, and no averaging over frames. Scans were converted to stacks using Scout-and-Scan Control System v.16.1.13038.43540 (Carl Zeiss, Oberkochen, Germany).

Three-dimensional models were constructed in Avizo v.2020.3.1 (Thermo Fisher Scientific, Waltham, MA, USA). Either the left or right trochanter was segmented, based on its accessibility, as some trochanters were obscured by glue. All voxels that were associated with the trochanters were identified using the brush tool in the segmentation editor. Surfaces were filled using the function ‘Fill - All slices under Selection’ after selecting the material in all three orientations and subsequently adding the fillers to the segmented trochanter. In some cases, voxels were manually selected to fix remaining gaps. Ambient occlusion was used to fill in the aspect of the trochanters where they contact the femur and the proximal gaps if present (Ege et al., 2020). The maximum distance used was 50, number of rays was 50, and the intensity range of the ‘Interactive Thresholding’ module was set from 0.5 to 1.0. After ambient occlusion, steps were largely as in Ege et al. (2020). Reduction to 18,000 faces was performed in the ‘Simplification Editor’ using default settings. Similarly, ‘Remesh Surface’ followed by ‘Smooth Surface’ were applied using default settings. The resulting surface data were exported as PLY data to be further processed in MeshLab v.2021.05 (Visual Computing Lab, ISTI, CNR). Data were archived open-access on MorphoSource (http://www.morphosource.org) under IDs 000801997, 000802002, 000802009, 000802015, 000802019, 000802025, 000802029, 000802033, 000802038, 000802042, 000802701, 000802705, 000802711, 000802715, 000802721, 000802725, 000802731, 000802735, 000802741, 000802745, 000802751, 000802755, 000802761, 000802765, 000802771, 000802775, 000802781, 000802785, 000802791, 000802795, 000802801, 000802805, 000806059, 000806063, 000806069, 000806073, 000806079, and 000806083.

### Image analysis

**Figure 2:**
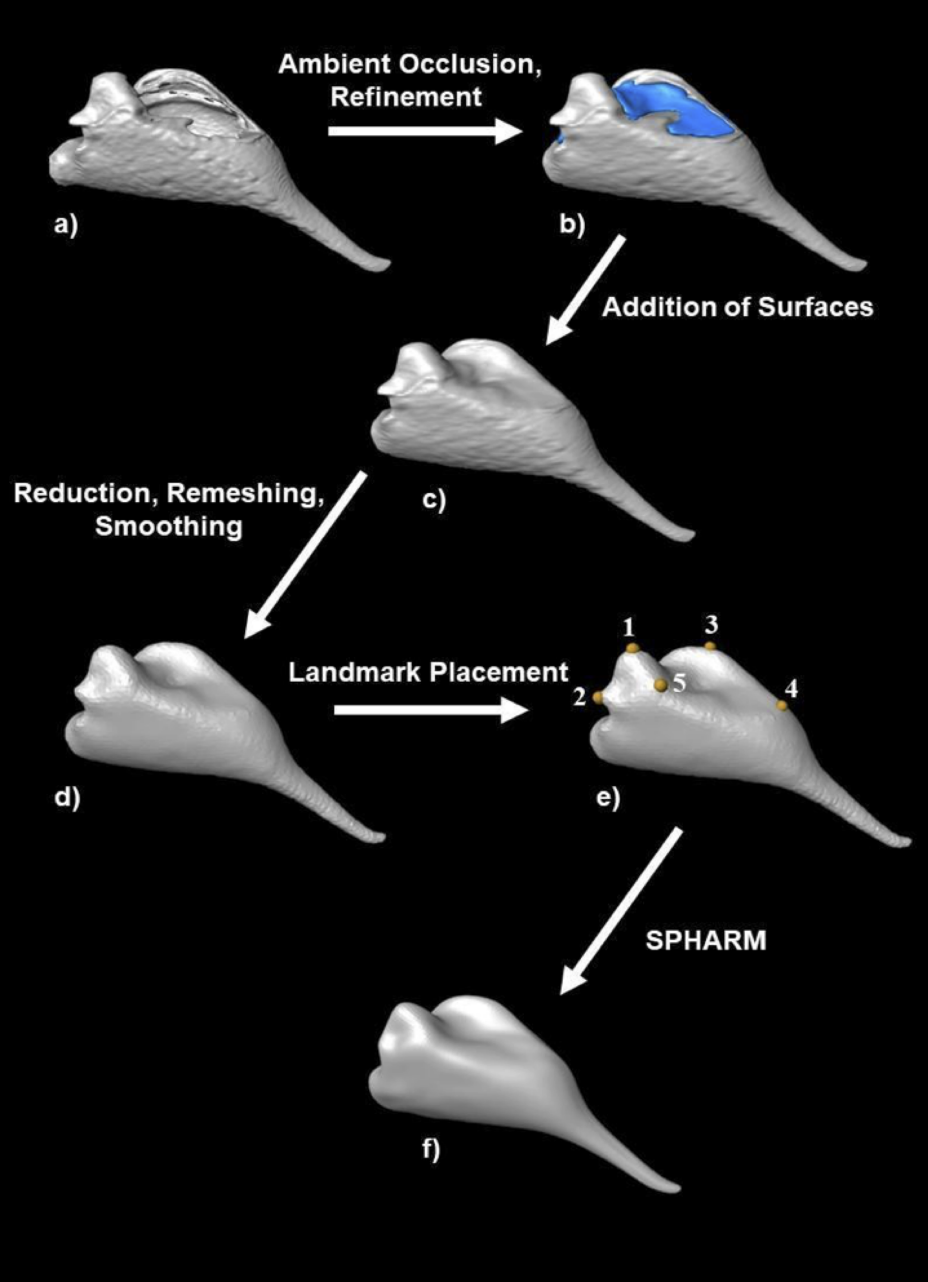
Workflow for 3D model reconstruction. Model resulted from segmentation in Avizo. All voxels were manually selected with the brush tool, enabling masking and resulting in a first crude 3D model (A). After filling in the gaps, ambient occlusion was performed to fill in the remaining dorsal gap (B) after which the surfaces were added and refined resulting in the total surface of the object (C). A first reduction in size, remeshing and smoothing was performed in Avizo with subsequent smoothing in MeshLab (D). Five landmarks were placed on the smoothed surface (E). Landmark data and smoothed surface data were fed into the SPHARM algorithm in MATLAB, resulting in the final 3D model (F). Shown is the left metatrochanter of *Choleva kocheri*.

MeshLab processing steps from Ege et al. (2020) were followed, with the modification that the pre-clean option needed to be applied in the ‘Surface reconstruction: Screened Poisson’ module in some trochanters (Fig. 2). Shape data were reduced to 4,452 vertices and 8,900 faces for each trochanter. Smoothed surfaces were exported as PLY data. Left trochanters were kept in the original orientation; right trochanters were imported in Avizo v.2020.3.1. (Thermo Fisher Scientific, Waltham, MA, USA) and mirrored. Then, five landmarks were manually placed on the smoothed surfaces in Avizo at homologous locations for each trochanter. These were chosen to reflect areas that are relatively constant, easily recognisable and comparable between and within meso- and metatrochanters. They include the trochanter-femoral joint which allows for flexibility of the segment relative to the femur (landmarks 1, 2) and aspects of the dorsal side of the metatrochanter (landmarks 3–5). Ventral aspects of the trochanters were not included because their interspecific variability made it impossible to find unambiguous, homologous landmarks.

Images were analysed using the SPHARM algorithmic framework (Shen & Makedon, 2006; Shen, Farid & McPeek, 2009) in MATLAB v.R2022a (The MathWorks, Natick, MA, USA). This includes a calculation of the centroids and a principal component analysis (PCA) on the covariance matrix of the SPHARM coefficients. Settings mentioned in Ege et al. (2020) were used, with the exception that only ten principal components were retained. Other settings were default. Meso- and metatrochanters were analysed separately, as well as in a combined dataset. This allowed us to easily infer if variation within metatrochanters was similar, greater or lower than within mesotrochanters and if these structures cluster together and can thus be considered morphologically similar. All steps are summarised in Fig. 2.

### Multivariate statistics

Principal component (PC) scores of the first four factors were visualised in scatterplots in R v.4.2.1 (R Foundation) and hypothetical 3D models of trochanters at extreme values for the PCs were generated in the SPHARM framework. To select PCs with biologically relevant variation, eigenvalues were compared with the mean eigenvalue and variation explained by eigenvalues was compared with random variation using the broken stick method (Jackson, 1993) in R v.4.2.1 (R Foundation).

### Calculation of the tempo of evolution

The ML tree constrained by the BI topology was imported into R v.4.2.1 (R Foundation) using the package *ape* v.5.6.2 (Paradis & Schliep, 2019). An ML estimate for Pagel’s κ (Pagel, 1994; Pagel, 1997; Pagel, 1999), together with the upper and lower boundaries for the 95% confidence interval, were calculated with the *motmot* v.2.1.3 package (Thomas & Freckleton, 2011). As input values for Pagel’s κ, the centroid and PC scores for the first four PCs were taken to reflect different aspects of shape variation across trochanter segments (Stefanini et al., 2021). Here, the centroid is interpreted as a measure of overall shape. Data used to calculate Pagel’s κ were generated by the combined SPHARM analysis of both meso- and metatrochanters. Estimates of κ were calculated separately for meso- and metatrochanters.

### Scripts

All R scripts are available as Supplementary Files 1.

## Results

### Molecular phylogenetics

**Figure 3:**
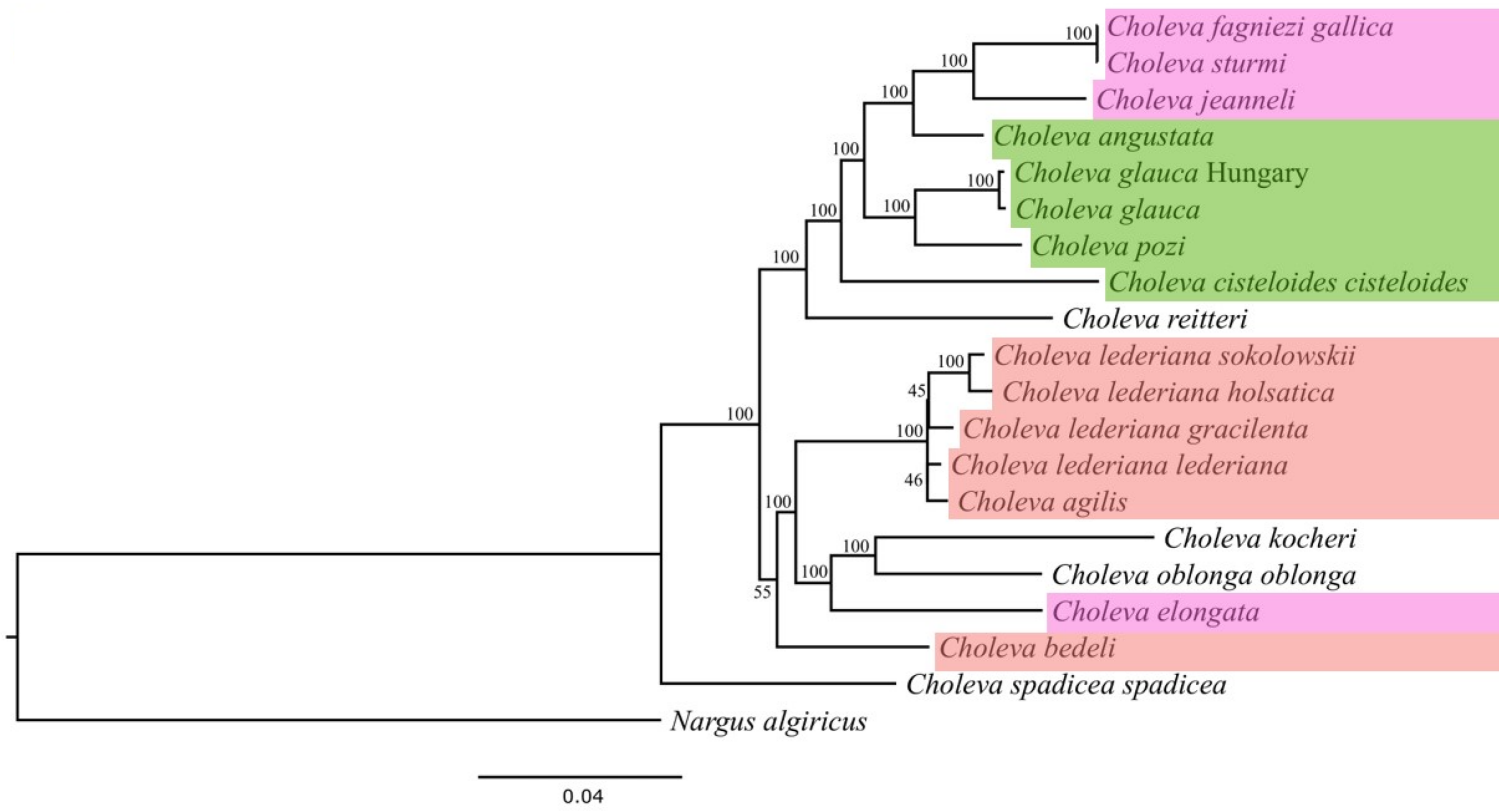
Maximum likelihood tree constrained by the Bayesian inference topology. Bootstrap values are given. Colours represent different species groups: purple, *sturmi*-group; green, *cisteloides*-group; salmon, *agilis*-group. All other species are single representatives of the following groups: *C. reitteri*, *reitteri*-group; *C. oblonga*, *oblonga*-group; *C. kocheri*, *kocheri*-group; *C. spadicea*, *Cholevopsis*-group. Because no other members of these groups were included, they were not coloured here.

In total, four phylogenetic trees were produced (Fig. S1). The time-calibrated BI tree produced a slightly different topology in comparison to the standard BI tree. For use in the downstream evolutionary analysis, two ML trees were produced, a stand-alone analysis and one that was constrained to the time-calibrated BI tree (Fig. 3).

**Figure 4:**
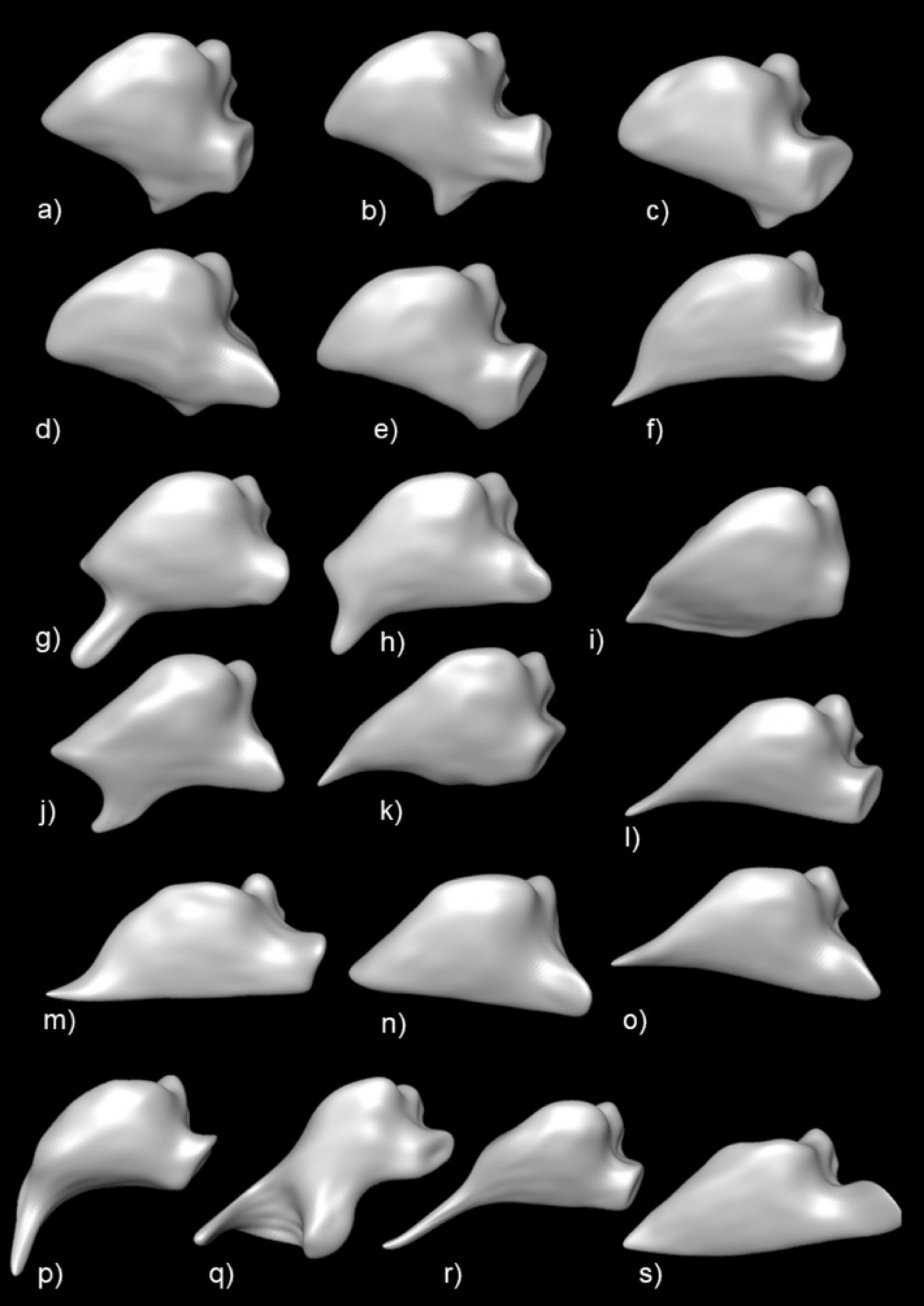
Male metatrochanter interspecific shape variation. All 3D spherical harmonic models have the same proportions and orientation. Species: (A) *Choleva (Choleva) agilis*; (B) *Choleva (Choleva) lederiana lederiana*; (C) *Choleva (Choleva) lederiana gracilenta*; (D) *Choleva (Choleva) lederiana sokolowskii*; (E) *Choleva (Choleva) lederiana holsatica*; (F) *Choleva (Choleva) bedeli*; (G) *Choleva (Choleva) glauca* (Hungary); (H) *Choleva (Choleva) glauca*; (I) *Choleva (Choleva) cisteloides cisteloides*; (J) *Choleva (Choleva) angustata*; (K) *Choleva (Choleva) pozi*; (L) *Choleva (Choleva) sturmi*; (M) *Choleva (Choleva) elongata*; (N) *Choleva (Choleva) jeanneli*; (O) *Choleva (Choleva) fagniezi gallica*; (P) *Choleva (Choleva) reitteri*; (Q) *Choleva (Choleva) oblonga oblonga*; (R) *Choleva (Choleva) kocheri*; (S) *Choleva (Cholevopsis) spadicea spadicea*.

### Spherical harmonics and principal components

**Figure 5:**
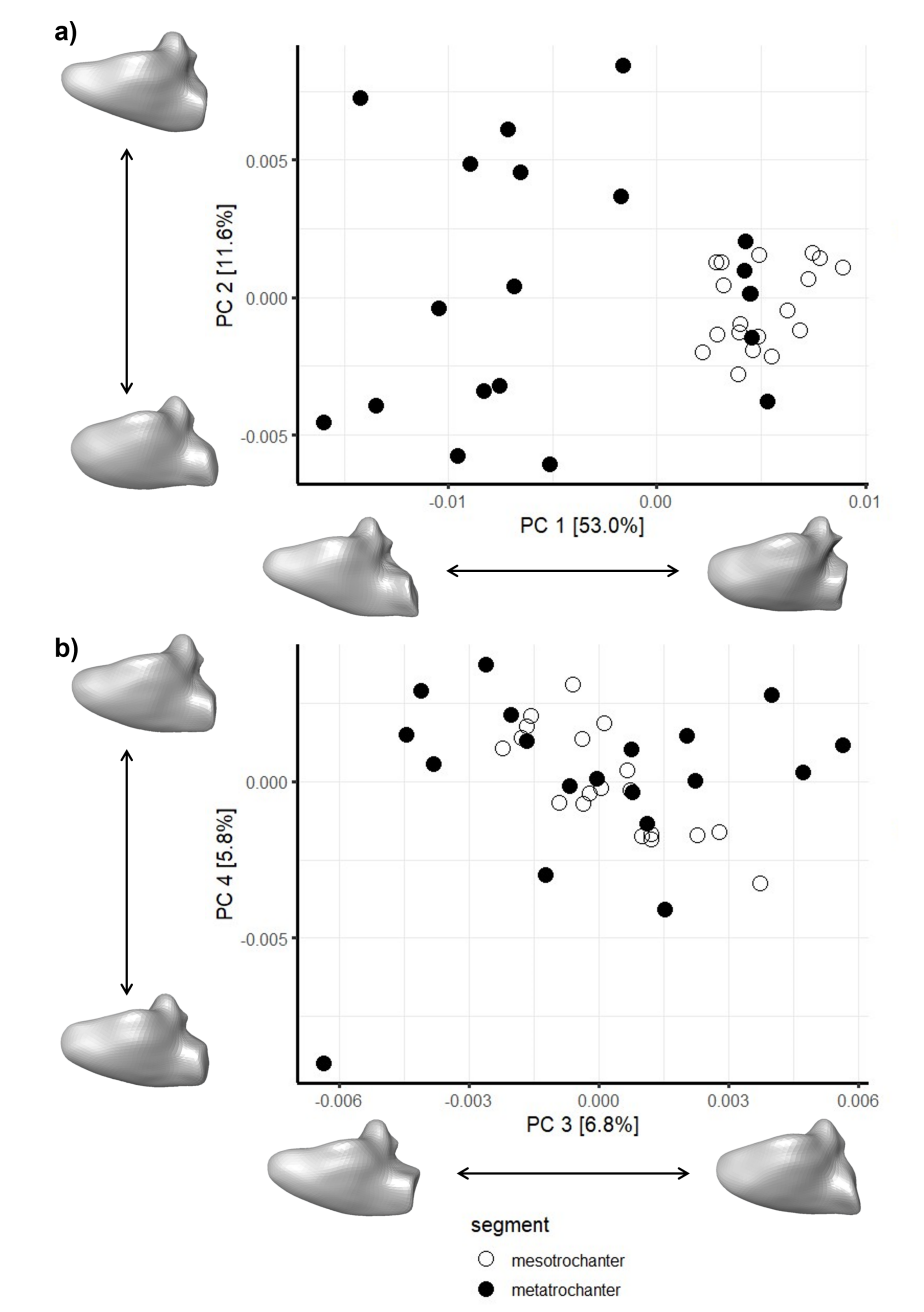
Scatterplots of the first four principal components. (A) Scores for principal components 1 and 2; (B) scores for principal components 3 and 4. Percent variation explained by each principal component is given within brackets on the axes. Points closer together are morphologically more similar. Shape variation on the principal component axes is shown with reconstructed spherical harmonic models for the mesotrochanters at values −2 and +2 at the extremes.

Three-dimensional models for 19 taxa were successfully obtained (Fig. 4). These clearly reflect the interspecific variability that have made the male metatrochanter such a valued taxonomic character in this genus. In contrast to the metatrochanter, variation in shape is very limited in the mesotrochanters (Fig. S2). This is also apparent when meso- and metatrochanters are analysed together in a PCA. The morphospace occupied by the metatrochanters was much larger than (and partly overlapping with) the morphospace occupied by the mesotrochanters (Fig. 5). For the metatrochanter, the eigenvalues for the first three principal components (PCs) were higher than the average eigenvalue, but only the first PC explained a higher percentage of variance than explained by the broken stick model. The second PC explained approximately equal amounts of variance. The first three PCs explain a cumulative variance of 74.2% (PC 1: 44.8%, PC 2: 18.6%, PC 3: 10.8%), while PC4 accounts for 6.5% of the variance. Negative values of PC 1 correspond with *Ch. agilis* and closely related species with a small proximo-ventral tooth and ventro-dorsal elongation, whereas positive PC 1 scores result in more proximo-distally elongated trochanters that are laterally flattened distally and without any distinct teeth ventrally. PC 2 mostly separates shapes based on variation between *Ch. sturmi* and *Ch. cisteloides* and related species. Negative values correspond with distally strongly tapered metatrochanters such as those of *Ch. sturmi*, *Ch. fagniezi gallica*, and *C. kocheri*. Positive values are largely rectangular in lateral view with a ventro-distal, laterally flattened wavy ridge, which has some similarities with the ventral ridge found in *Ch. cisteloides*. On PC 3, negative values correspond with the metatrochanter of *Ch. oblonga* that has a cup-like shape distally but is less elongated in proximo-distal direction. Positive values reflect trochanters with a large ventro-distal tooth like in *Ch. angustata* and *Ch. glauca*. Finally, negative scores of PC 4 resemble a trochanter with a waved ventral ridge, most reminiscent of that found in *C. cisteloides*. Positive values of this PC follow a distally tapered shape with a shallow ridge ventrally, comparable to *C. pozi*.

### Tempo of evolution

Values of Pagel’s κ were generally below 0.5 for both meso- and metatrochanter (Table 1). The only value with an ML estimate of >0.5 was that of the metatrochanter centroid, with a lower 95% confidence interval (CI) boundary of approximately 0.28. For the mesotrochanter, ML estimates of κ were between 0.0 and 0.17, with upper CI estimates not exceeding 0.4. Especially values for PC scores on PCs 2, 3, and 4 were extremely low. For the metatrochanter, the PC 1 score had the lowest value (ML estimate of κ = 10^−8^), whereas the other PC scores had ML estimates for κ between 0.1 and 0.2.

## Discussion

Although the limited availability of fresh material restricted our study to 19 (sub)species, around one quarter (Perreau, 2000) of the total diversity of *Choleva*, we expect that we sampled sufficient morphological and taxonomic diversity to reveal evolutionary patterns. The estimates of Pagel’s κ for the metatrochanter centroid suggest that the overall shape of the metatrochanter has evolved in a continuous fashion over time. This would suggest an evolutionary process that is driven primarily by intraspecific SAC or CFC. However, specific aspects of its shape, reflected by the PC scores (particularly PC1, which correlates with tooth shape), show a signature of punctuated evolution, which suggests that these features play a role in mate recognition and may have evolved by reinforcement during speciation and/or reproductive character displacement immediately after speciation.

**Table 1:** All Pagel’s kappa values (for meso and metatrochanters).

**Variable**	**ML**κ	**95% CI Lower**κ	**95% CI Upper**κ
**Mesotrochanter**			
centroid	0.1359206	0.00000001	0.2992264
PC 1 score	0.1663721	0.00000001	0.3820837
PC 2 score	0.00000001	0.00000001	0.2031384
PC 3 score	0.00000001	0.00000001	0.2284022
PC 4 score	0.00000001	0.00000001	0.1539075
**Metatrochanter**			
centroid	0.6362069	0.2828399	0.8350545
PC 1 score	0.00000001	0.00000001	0.1629484
PC 2 score	0.2062542	0.00000001	0.3776448
PC 3 score	0.1020028	0.00000001	0.275069
PC 4 score	0.1772936	0.00000001	0.3232623

**Notes.**

CI, confidence interval.

It is not unexpected that different features of the same organ evolve under different selection pressures. For example, in the *Drosophila repleta* species group, aedeagus size and specific features of aedeagus shape follow models of speciational and gradual change, respectively, (Stefanini et al., 2021), similar to what we find in *Choleva*. Furthermore, Arbuthnott et al. (2010) found that male courtship behaviours in *Timema* stick insects evolved in a punctuated mode over time, whereas male external genitalia evolved continuously over time, illustrating that different selection pressures shaped the evolutionary trajectories of these characters.

**Figure 6:**
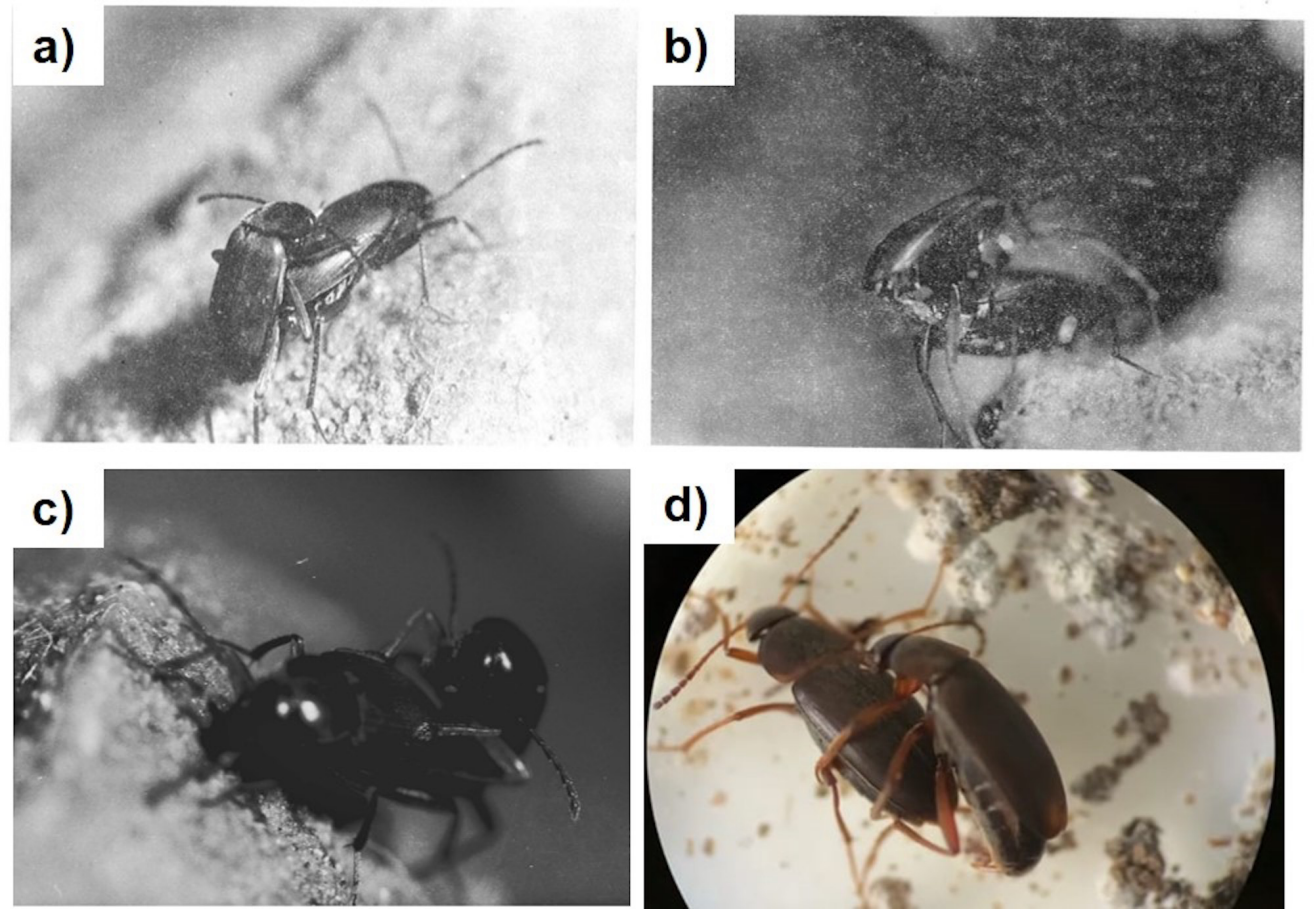
Mating positions of *Choleva* species. (A, B) Unknown *Choleva* species, *in situ*, Ramioul Cave (*after Hubart, 1973)*; (C) *Choleva spadicea spadicea*, *in situ*, Ramioul Cave (after Thieren & Dethier, 2006); (D) *Choleva lederiana gracilenta*, in the lab (photo by M.S.).

Unfortunately, we have currently no knowledge of the exact role of the metatrochanter during courtship and mating in *Choleva*. The few published descriptions and images of copulation in the genus (Benick, 1937; Deleurance, 1959; Zwick, 1966; Hubart, 1973; Thieren & Dethier, 2006) suggest that the metatrochanter connects with the tip of the female elytra and/or of the abdomen (Fig. 6), but no in-depth behavioural analysis is available to provide more detail. In theory, it is possible that the shape of the metatrochanter is detected by sensilla on the parts of the female that come into contact with it, and that it is important in courtship and/or in mate recognition. Alternatively, one could also imagine that the metatrochanter is used to pry open the tip of the abdomen and/or the (species-specifically shaped) female genital tergite, and thus that it plays a role in sexually antagonistic coevolution. The elongated elytral apices that the females of some species, specifically within the ‘*sturmi*-group’, possess, could be interpreted as a defensive character. However, without more detailed observations of copulatory courtship or resistance behaviour, evidence for CFC or SAC is therefore at best circumstantial. Also, our taxon sampling included only four taxa from the ‘*sturmi*-group’, which precluded an analysis of coevolution between male metatrochanter and female elytral apex shapes. More insight may be obtained by a full analysis of the entire ‘*sturmi*-group’ and by comparison with other insects in which the male metatrochanter is similarly diverse. Species-specific differences in metatrochanter morphology are also diagnostic in other insect groups (*e.g.*, Mohamedsaid, 2010; Xie & Liu, 2015; Van Steenis et al., 2016; Mengual, 2018; Ramage, Charlat & Mengual, 2018; Bezděk, 2020; Duarte & Grossi, 2020), and future studies of these taxa may reveal similarities in mating behaviour that, by analogy, could be applied to *Choleva* as well.

To compare the morphospace occupied by the male metatrochanter, we also analysed the (sexually and interspecifically monomorphic) male mesotrochanter. Interestingly, Pagel’s κ values for the mesotrochanter were generally very low. When values of Pagel’s κ are close to zero, traits evolve in a tempo that is decoupled from branch length, *i.e.,* in a pattern of punctuated evolution (Pagel, 1994; Pagel, 1997; Pagel, 1999). However, compared to the morphology of metatrochanters (Fig. 5, closed symbols), the variation in shape of the mesotrochanter is very limited (Fig. 5, open symbols). We suggest that the two segments may be developmentally coupled, and the evolutionary pressures that the metatrochanter is subjected to, ‘leak’ into the development of the mesotrochanter as well, albeit in a much-reduced manner. A future test of this hypothesis would be to perform a similar study on the female trochanters.

Finally, our phylogenetic reconstruction has implications for the intrageneric systematics of *Choleva*. *Ch. agilis* and all subspecies of *Ch. lederiana* form a polytomy, confirming the close relationship within this complex (Jeannel, 1936; Schilthuizen, 1990; Růžička & Vavra, 2003). However, *Ch. bedeli*, which Jeannel (1936) also placed in the close phylogenetic vicinity of *Ch. agilis*, appears distantly related. Similarly, we find that *C. elongata* Paykull 1798 is not part of the *sturmi* group in which it has been traditionally placed (Jeannel, 1936).

## Conclusions

Our study showed that the morphological diversity in a non-genitalic, sexually dimorphic contact character, the male metatrochanter in *Choleva*, can be successfully quantified and evolutionarily analyzed by micro-CT-scanning and multivariate statistical analysis of spherical harmonics parameters. Our analysis resulted in a clear signal of divergent evolution in which both gradual and punctuated change played a role. Although the rate of change was much greater in the metatrochanter, we found that the mesotrochanter is subject to similar evolutionary trends as well. Provisionally, we explain this as a by-product of the selection that primarily drives the metatrochanter shape, caused by shared developmental mechanisms. The behavioural mechanisms that drive the divergence of the male metatrochanter remain unknown until we have further information on the exact manner in which it connects with the female during courtship and mating. Conceivably, intraspecifically, either cryptic female choice or sexually antagonistic coevolution, or both, might play a role in the gradual changes in morphology. Interspecifically, speciation by reinforcement and/or post-speciation reproductive character displacement are likely candidate processes for the punctuational evolutionary change.

## Supplemental Information

10.7717/peerj.21266/supp-1 Supplemental Information 1 All specimens we used, their collection numbers, origin, and the sequences we obtained (with Genbank numbers)

10.7717/peerj.21266/supp-2 Supplemental Information 2 Primer combinations and corresponding annealing temperatures

10.7717/peerj.21266/supp-3 Supplemental Information 3 Spherical harmonic data for all specimens and both trochanters

10.7717/peerj.21266/supp-4 Supplemental Information 4 Phylogenies produced during this study A and C show a Bayesian tree, whereas B and D are a visualization of a maximum-likelihood tree. A clock model was used in C and this tree was used as a constraint in the building of D. Three subgroups within the genus have been marked: *agilis* (red), *sturmi* (pink) and *cisteloides* (green). *Nargus algiricus* was used as an outgroup.

10.7717/peerj.21266/supp-5 Supplemental Information 5 Male mesotrochanter shape variation All 3D spherical harmonic models have the same proportions and orientation. Species: (A) *Choleva (Choleva) agilis*; (B) *Choleva (Choleva) lederiana lederiana*; (C) *Choleva (Choleva) lederiana gracilenta*; (D) *Choleva (Choleva) lederiana sokolowskii*; (E) *Choleva (Choleva) lederiana holsatica*; (F) *Choleva (Choleva) bedeli*; (G) *Choleva (Choleva) glauca* (Hungary); (H) *Choleva (Choleva) glauca*; (I) *Choleva (Choleva) cisteloides cisteloides*; (J) *Choleva (Choleva) angustata*; (K) *Choleva (Choleva) pozi*; (L) *Choleva (Choleva) sturmi*; (M) *Choleva (Choleva) elongata*; (N) *Choleva (Choleva) jeanneli*; (O) *Choleva (Choleva) fagniezi gallica*; (P) *Choleva (Choleva) reitteri*; (Q) *Choleva (Choleva) oblonga oblonga*; (R) *Choleva (Choleva) kocheri*; (S) *Choleva (Cholevopsis) spadicea spadicea*. Colours refer to different species groups: (A-F) salmon, *agilis*-group; (G-K) green, *cisteloides*-group; (L-O) pink, *sturmi*-group. All other species are single representatives of their respective groups: (P) *reitteri*-group, (Q) *oblonga*-group, (R) *kocheri*-group, (S) *Cholevopsis*-group.

10.7717/peerj.21266/supp-6 Supplemental Information 6 R scripts used in this study

## Additional Information and Declarations

### Data Availability

The following information was supplied regarding data availability: The code is available in the Supplemental Files. The sequences are accessible via GenBank: KM451525, PQ817991–PQ818016, PQ834293–PQ834310, PQ835050–PQ835099, and PQ849690–PQ849728.
