# Supplementary material for: Evolutionary divergence in a non-genitalic sexual contact character in the beetle genus Choleva
Source: PeerJ. 2026 Jun 10;14:e21266. doi: 10.7717/peerj.21266 (PMC13264280; doi:10.7717/peerj.21266)
Supplement: Supplemental Information 3 [file peerj-14-21266-s003.docx]

Supplementary Table 3: Spherical Harmonic data for combined meso- and metatrochanter data

| **Sample ID** | **Species** | **Trochanter** | **Group** | **Centroid** | **PC 1 score** | **PC 2 score** | **PC 3 score** | **PC 4 score** |
| --- | --- | --- | --- | --- | --- | --- | --- | --- |
| 15.GL.41 | *Choleva glauca* | mesotrochanter | *cisteloides* | 7281.636153 | 0.002864 | 0.001269 | 0.003727 | -0.003244 |
| 15.GL.41 | *Choleva glauca* | metatrochanter | *cisteloides* | 8680.120946 | -0.007556 | -0.003207 | 0.004736 | 0.000303 |
| 16.EL.64 | *Choleva elongata* | mesotrochanter | *sturmi* | 8783.052376 | 0.003217 | 0.000457 | 0.000989 | -0.001748 |
| 16.EL.64 | *Choleva elongata* | metatrochanter | *sturmi* | 10469.93999 | -0.006554 | 0.004564 | 0.00111 | -0.001339 |
| 17.OB.28 | *Choleva oblonga oblonga* | mesotrochanter | *oblonga* | 8762.990368 | 0.004888 | 0.001538 | 0.00278 | -0.001603 |
| 17.OB.28 | *Choleva oblonga oblonga* | metatrochanter | *oblonga* | 13098.22408 | -0.015969 | -0.004547 | -0.006352 | -0.008989 |
| 18.SP.34 | *Choleva spadicea spadicea* | mesotrochanter | *Cholevopsis* | 7842.678165 | 0.002170 | -0.001972 | -0.001783 | 0.001413 |
| 18.SP.34 | *Choleva spadicea spadicea* | metatrochanter | *Cholevopsis* | 10194.27982 | -0.008976 | 0.004862 | 0.001538 | -0.004075 |
| 26.AG.96 | *Choleva agilis* | mesotrochanter | *agilis* | 7408.284113 | 0.007235 | 0.000663 | -0.000208 | -0.000372 |
| 26.AG.96 | *Choleva agilis* | metatrochanter | *agilis* | 8108.067471 | 0.005282 | -0.003787 | -0.003815 | 0.000551 |
| 30.RE.12 | *Choleva reitteri* | mesotrochanter | *reitteri* | 7846.807723 | 0.003943 | -0.001249 | -0.001568 | 0.002084 |
| 30.RE.12 | *Choleva reitteri* | metatrochanter | *reitteri* | 11009.04302 | -0.013480 | -0.003927 | 0.004 | 0.002764 |
| 31.ST.PAL | *Choleva sturmi* | mesotrochanter | *sturmi* | 7812.555145 | 0.004846 | -0.001421 | -0.00166 | 0.00178 |
| 31.ST.PAL | *Choleva sturmi* | metatrochanter | *sturmi* | 9806.537848 | -0.007145 | 0.006112 | -0.002019 | 0.002125 |
| 33.KO.PER | *Choleva kocheri* | mesotrochanter | *kocheri* | 6720.797336 | 0.003096 | 0.001278 | 0.002292 | -0.001722 |
| 33.KO.PER | *Choleva kocheri* | metatrochanter | *kocheri* | 9946.121643 | -0.014241 | 0.007259 | -0.002597 | 0.003723 |
| 40.FA.PAL | *Choleva fagniezi gallica* | metatrochanter | *sturmi* | 9746.135154 | -0.001650 | 0.008419 | 0.00079 | -0.000334 |
| 43.AN.92 | *Choleva fagniezi gallica* | mesotrochanter | *sturmi* | 7185.754056 | 0.005488 | -0.002154 | -0.0006 | 0.003109 |
| 43.AN.92 | *Choleva angustata* | metatrochanter | *cisteloides* | 9325.301633 | -0.005130 | -0.006044 | 0.002221 | 0.000011 |
| 45.FA.PAL | *Choleva angustata* | mesotrochanter | *cisteloides* | 8333.4975 | 0.006230 | -0.000454 | 0.000655 | 0.00035 |
| 46.LELE.86 | *Choleva lederiana lederiana* | metatrochanter | *agilis* | 8087.811325 | 0.004571 | -0.001451 | -0.001237 | -0.002981 |
| 46.LELE.86 | *Choleva lederiana lederiana* | mesotrochanter | *agilis* | 7360.090818 | 0.006878 | -0.001193 | -0.000902 | -0.000693 |
| 49.CI.16 | *Choleva cisteloides cisteloides* | mesotrochanter | *cisteloides* | 7365.581797 | 0.003978 | -0.000964 | 0.000728 | -0.000263 |
| 49.CI.16 | *Choleva cisteloides cisteloides* | metatrochanter | *cisteloides* | 9218.906541 | -0.008302 | -0.003402 | -0.004447 | 0.001511 |
| 50.LESO.TH | *Choleva lederiana sokolowskii* | mesotrochanter | *agilis* | 7775.500164 | 0.007450 | 0.001629 | 0.001226 | -0.001846 |
| 51.BE.TA | *Choleva bedeli* | mesotrochanter | *agilis* | 6443.998781 | 0.004437 | 0.000128 | 0.000059 | -0.000196 |
| 51.BE.TA | *Choleva bedeli* | metatrochanter | *agilis* | 8385.331329 | -0.010452 | -0.000376 | 0.002027 | 0.001462 |
| 53.LEHO.TH | *Choleva lederiana holsatica* | mesotrochanter | *agilis* | 8590.517588 | 0.008921 | 0.001104 | -0.000355 | -0.000707 |
| 53.LEHO.TH | *Choleva lederiana holsatica* | metatrochanter | *agilis* | 8984.657116 | 0.004229 | 0.002032 | -0.001651 | 0.001308 |
| 55.LEGR.PL15 | *Choleva lederiana gracilenta* | mesotrochanter | *agilis* | 7007.489887 | 0.007787 | 0.001441 | 0.001219 | -0.001693 |
| 55.LEGR.PL15 | *Choleva lederiana gracilenta* | metatrochanter | *agilis* | 7160.295735 | 0.004513 | 0.000128 | -0.000673 | -0.000148 |
| 56.GL.HU4 | *Choleva glauca* “Hungary” | mesotrochanter | *cisteloides* | 6758.735354 | 0.004576 | -0.001928 | -0.000372 | 0.001349 |
| 56.GL.HU4 | *Choleva glauca* “Hungary” | metatrochanter | *cisteloides* | 8422.370334 | -0.009578 | -0.005738 | 0.005647 | 0.001157 |
| 60.LESO.TH | *Choleva lederiana sokolowskii* | metatrochanter | *agilis* | 7844.096515 | 0.004208 | 0.000972 | -0.000031 | 0.000108 |
| 64.PO.4245 | *Choleva pozi* | mesotrochanter | *cisteloides* | 7049.125697 | 0.003883 | -0.002786 | -0.002221 | 0.001058 |
| 64.PO.4245 | *Choleva pozi* | metatrochanter | *cisteloides* | 8831.66082 | -0.006820 | 0.000392 | -0.004115 | 0.00289 |
| 65.JE.28 | *Choleva jeanneli* | mesotrochanter | *sturmi* | 8548.224522 | 0.002867 | -0.001332 | 0.000117 | 0.001876 |
| 65.JE.28 | *Choleva jeanneli* | metatrochanter | *sturmi* | 9716.831734 | -0.001705 | 0.003687 | 0.000745 | 0.001025 |
