# Supplementary figures and images for: Evolutionary divergence in a non-genitalic sexual contact character in the beetle genus Choleva
Source: PeerJ. 2026 Jun 10;14:e21266. doi: 10.7717/peerj.21266 (PMC13264280; doi:10.7717/peerj.21266)

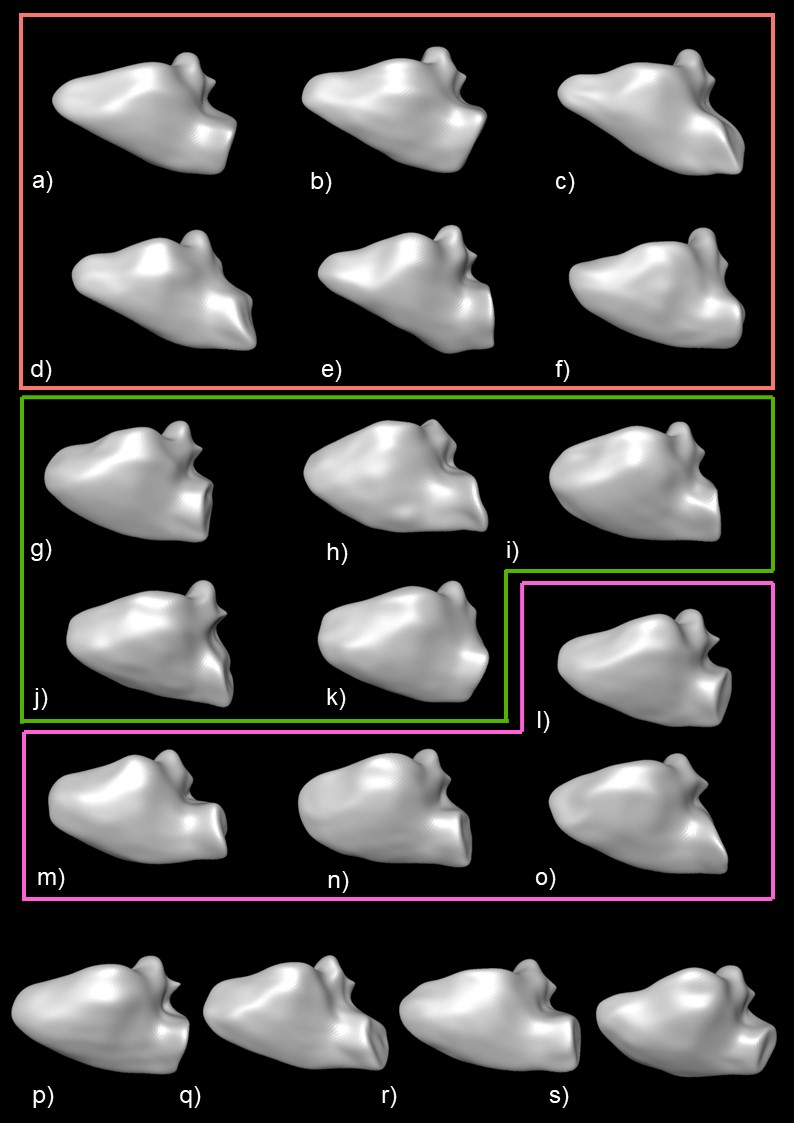

Supplement: Supplemental Information 5 — All 3D spherical harmonic models have the same proportions and orientation. Species: (A) Choleva (Choleva) agilis; (B) Choleva (Choleva) lederiana lederiana; (C) Choleva (Choleva) lederiana gracilenta; (D) Choleva (Choleva) lederiana sokolowskii; (E) Choleva (Choleva) lederiana holsatica; (F) Choleva (Choleva) bedeli; (G) Choleva (Choleva) glauca (Hungary); (H) Choleva (Choleva) glauca; (I) Choleva (Choleva) cisteloides cisteloides; (J) Choleva (Choleva) angustata; (K) Choleva (Choleva) pozi; (L) Choleva (Choleva) sturmi; (M) Choleva (Choleva) elongata; (N) Choleva (Choleva) jeanneli; (O) Choleva (Choleva) fagniezi gallica; (P) Choleva (Choleva) reitteri; (Q) Choleva (Choleva) oblonga oblonga; (R) Choleva (Choleva) kocheri; (S) Choleva (Cholevopsis) spadicea spadicea. Colours refer to different species groups: (A-F) salmon, agilis-group; (G-K) green, cisteloides-group; (L-O) pink, sturmi-group. All other species are single representatives of their respective groups: (P) reitteri-group, (Q) oblonga-group, (R) kocheri-group, (S) Cholevopsis-group. [file peerj-14-21266-s005.jpg]

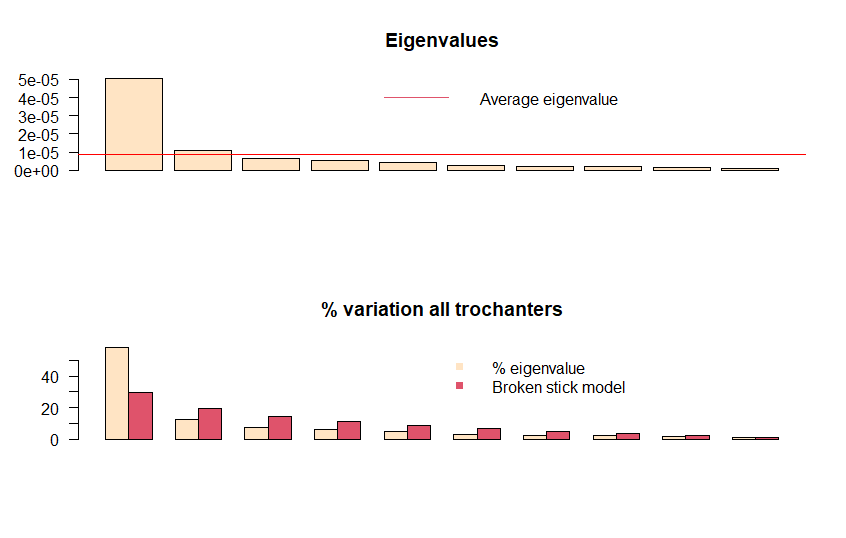

Supplement: Supplemental Information 6 [file peerj-14-21266-s006.zip › R analyses/Data analysis 16jul2022/broken stick method all trochanters.png]

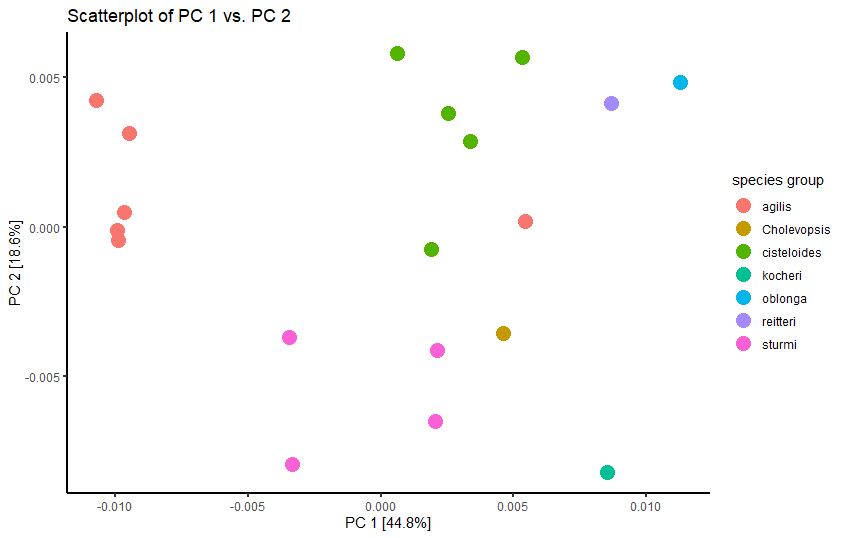

Supplement: Supplemental Information 6 [file peerj-14-21266-s006.zip › R analyses/Data analysis 16jul2022/pca metatrochanter pc1 vs pc2.png]

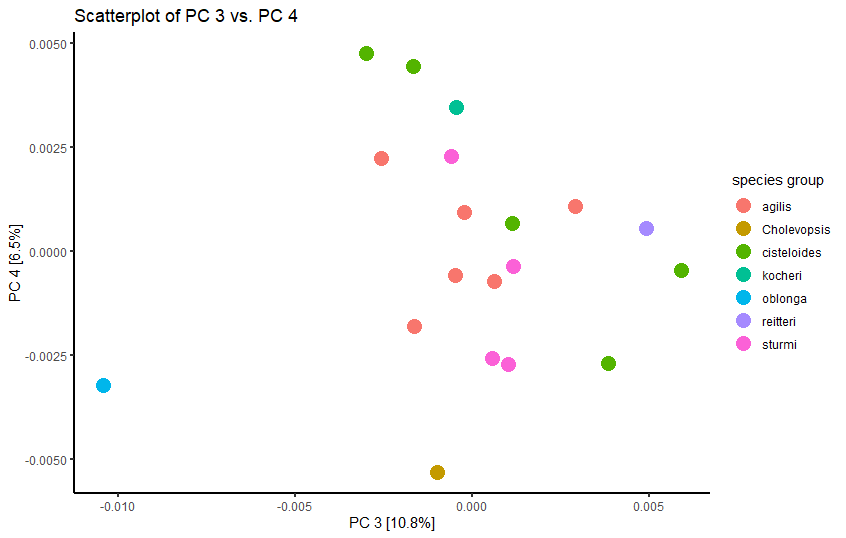

Supplement: Supplemental Information 6 [file peerj-14-21266-s006.zip › R analyses/Data analysis 16jul2022/pca metatrochanter pc3 vs pc4.png]

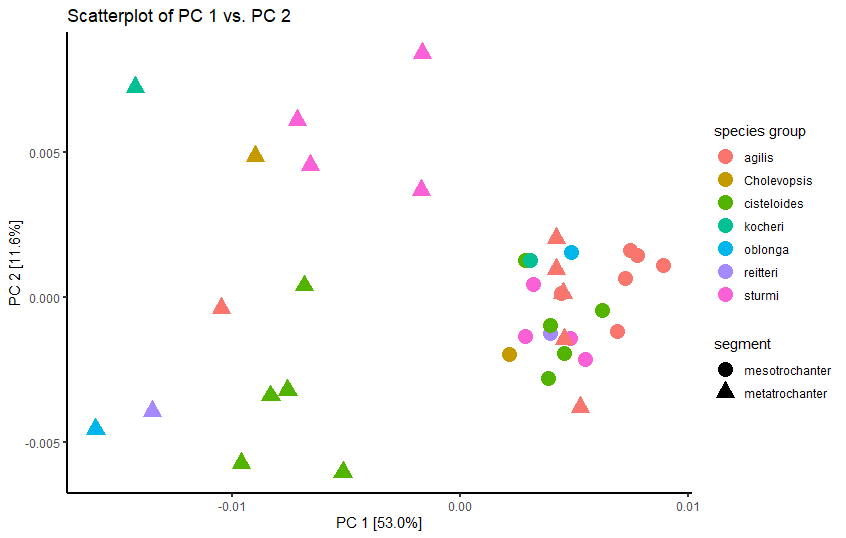

Supplement: Supplemental Information 6 [file peerj-14-21266-s006.zip › R analyses/Data analysis 16jul2022/pca pc1 vs pc2 all trochanters.png]

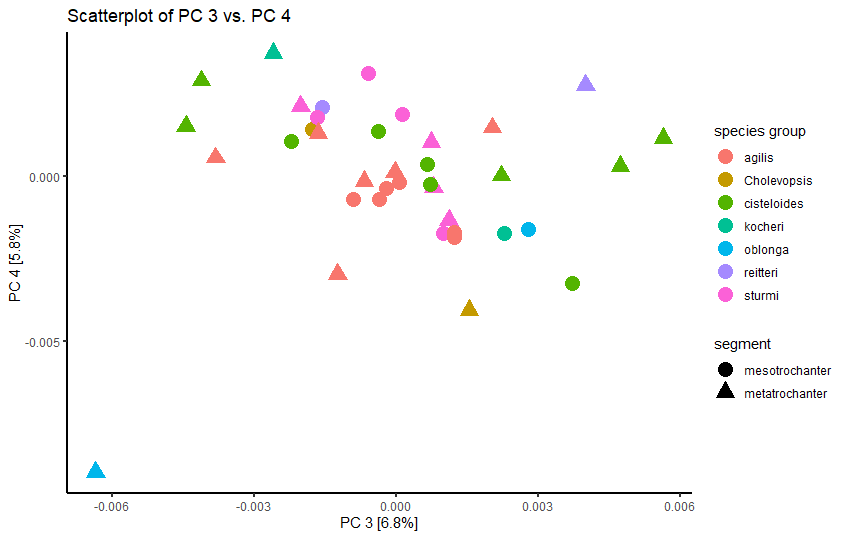

Supplement: Supplemental Information 6 [file peerj-14-21266-s006.zip › R analyses/Data analysis 16jul2022/pca pc3 vs pc4 all trochanters.png]

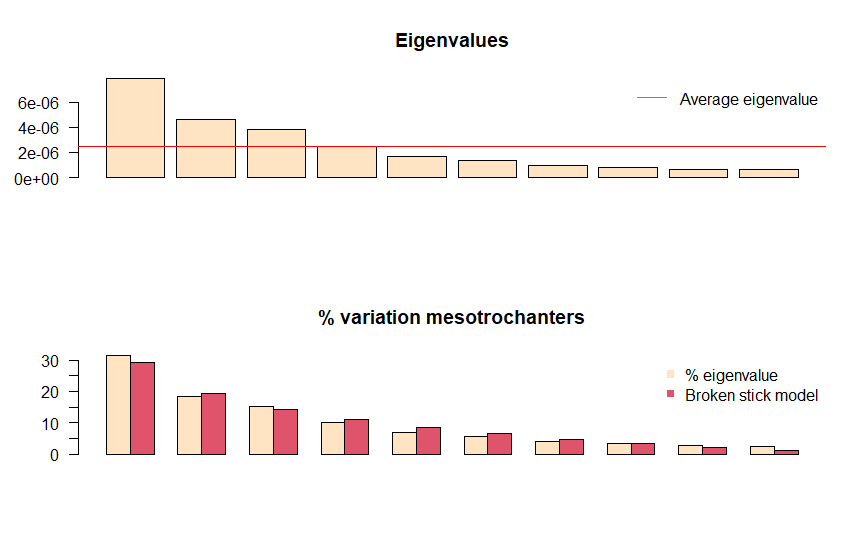

Supplement: Supplemental Information 6 [file peerj-14-21266-s006.zip › R analyses/Data analysis 18jul2022/broken stick meso.png]

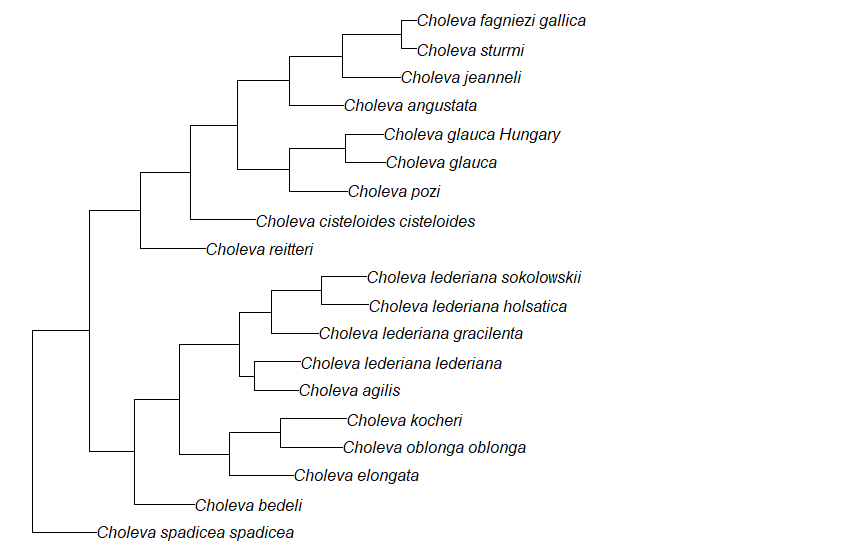

Supplement: Supplemental Information 6 [file peerj-14-21266-s006.zip › R analyses/Data analysis 18jul2022/kappa meso centroid.png]

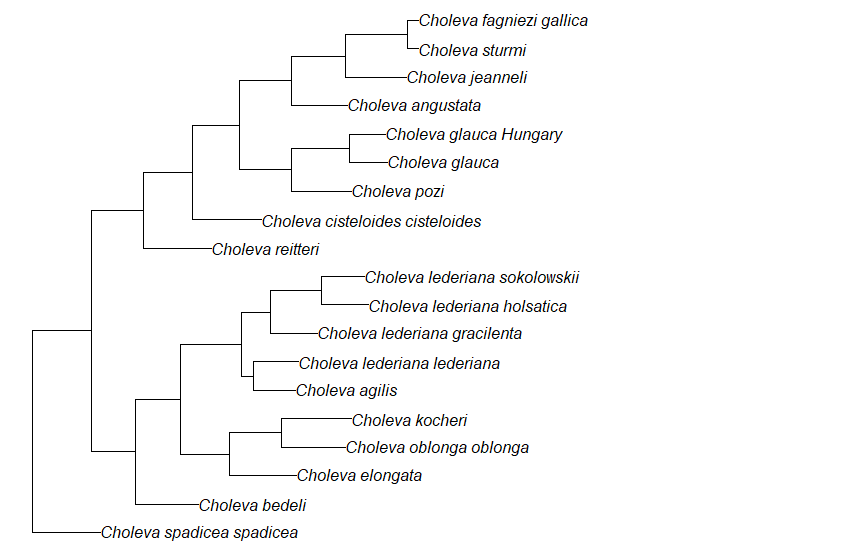

Supplement: Supplemental Information 6 [file peerj-14-21266-s006.zip › R analyses/Data analysis 18jul2022/kappa meso pc1.png]

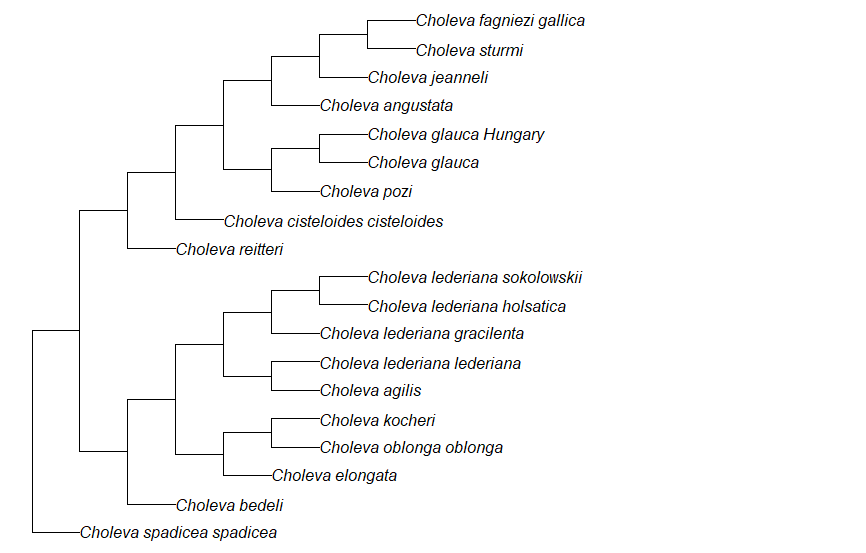

Supplement: Supplemental Information 6 [file peerj-14-21266-s006.zip › R analyses/Data analysis 18jul2022/kappa meso pc2.png]

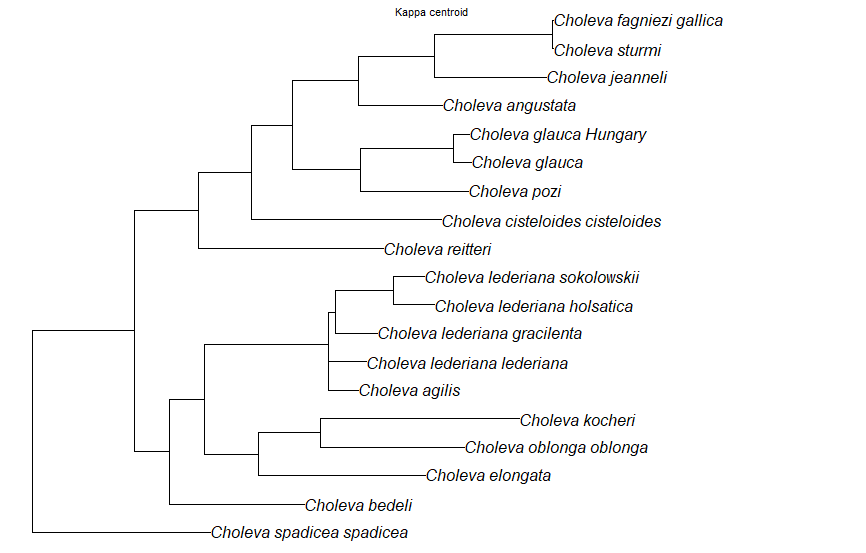

Supplement: Supplemental Information 6 [file peerj-14-21266-s006.zip › R analyses/Data analysis 18jul2022/kappa meta centroid.png]

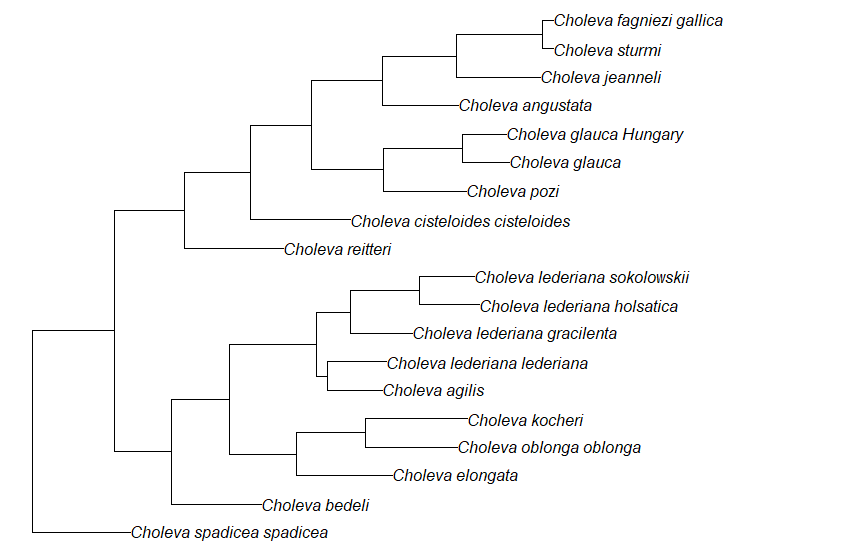

Supplement: Supplemental Information 6 [file peerj-14-21266-s006.zip › R analyses/Data analysis 18jul2022/kappa meta pc2.png]

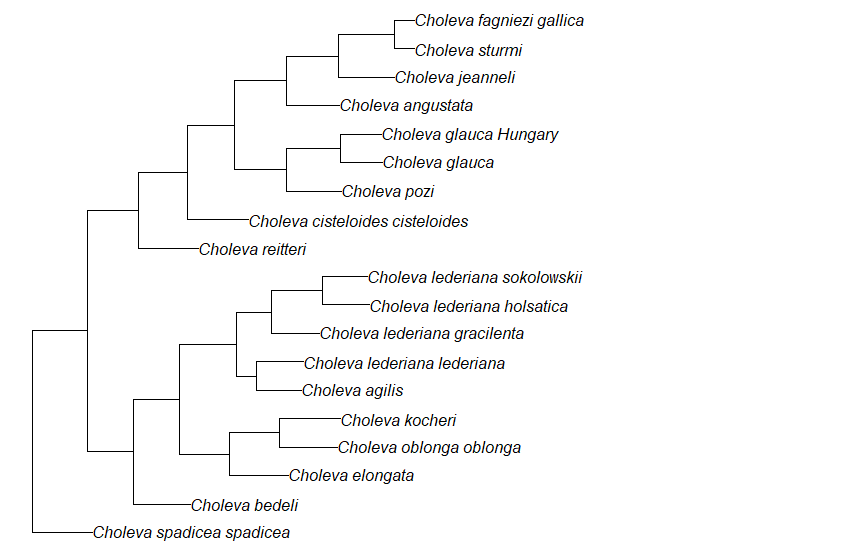

Supplement: Supplemental Information 6 [file peerj-14-21266-s006.zip › R analyses/Data analysis 18jul2022/kappa meta pc3.png]

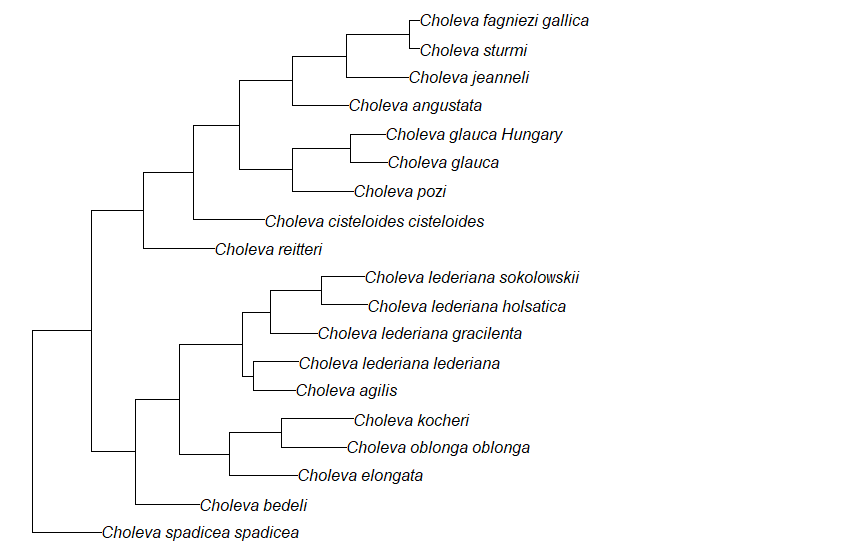

Supplement: Supplemental Information 6 [file peerj-14-21266-s006.zip › R analyses/Data analysis 18jul2022/kappa meta pc4.png]

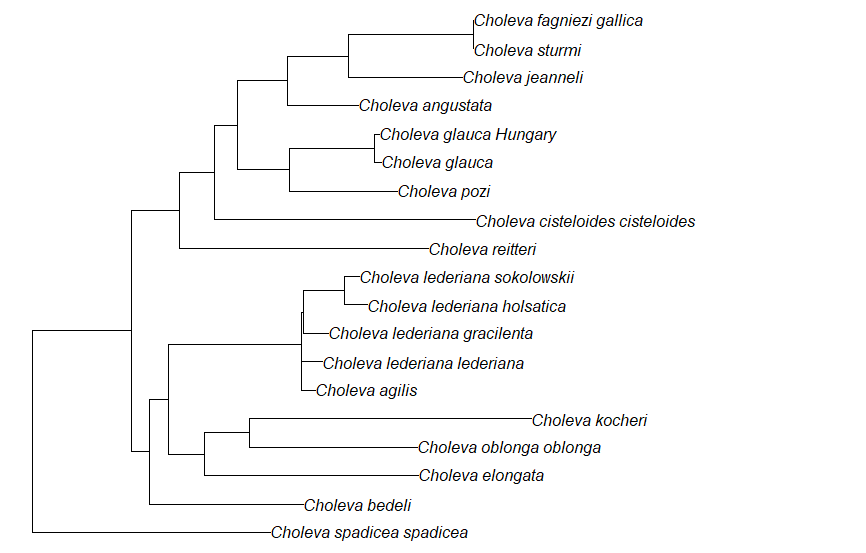

Supplement: Supplemental Information 6 [file peerj-14-21266-s006.zip › R analyses/Data analysis 18jul2022/meso original phylogeny.png]

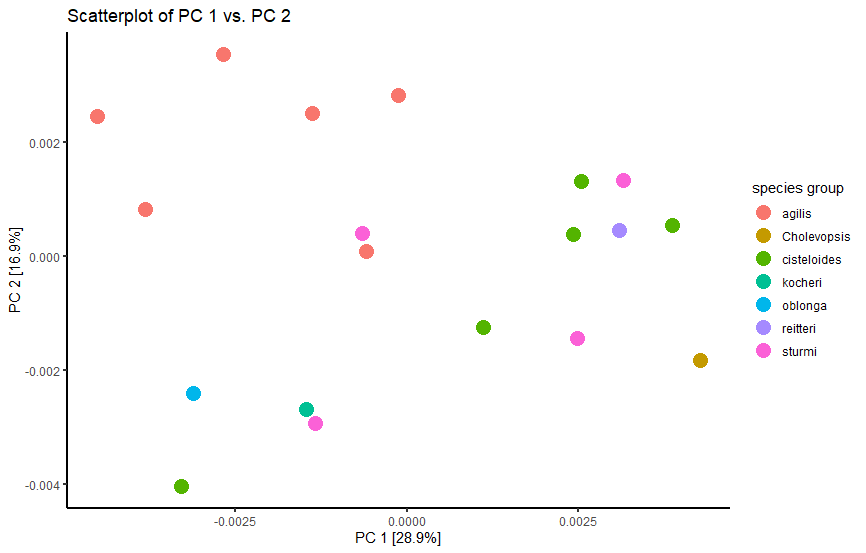

Supplement: Supplemental Information 6 [file peerj-14-21266-s006.zip › R analyses/Data analysis 18jul2022/pc1 vs pc2 meso.png]

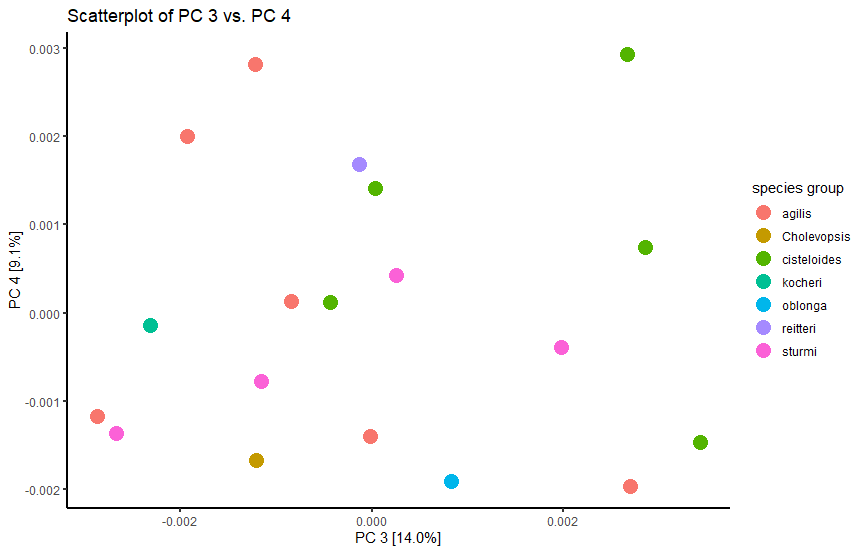

Supplement: Supplemental Information 6 [file peerj-14-21266-s006.zip › R analyses/Data analysis 18jul2022/pc3 vs pc4 meso.png]
