# Supplementary material for: Evolutionary divergence in a non-genitalic sexual contact character in the beetle genus Choleva
Source: PeerJ. 2026 Jun 10;14:e21266. doi: 10.7717/peerj.21266 (PMC13264280; doi:10.7717/peerj.21266)
Supplement: Supplemental Information 4 — A and C show a Bayesian tree, whereas B and D are a visualization of a maximum-likelihood tree. A clock model was used in C and this tree was used as a constraint in the building of D. Three subgroups within the genus have been marked: agilis (red), sturmi (pink) and cisteloides (green). Nargus algiricus was used as an outgroup. [file peerj-14-21266-s004.pdf]

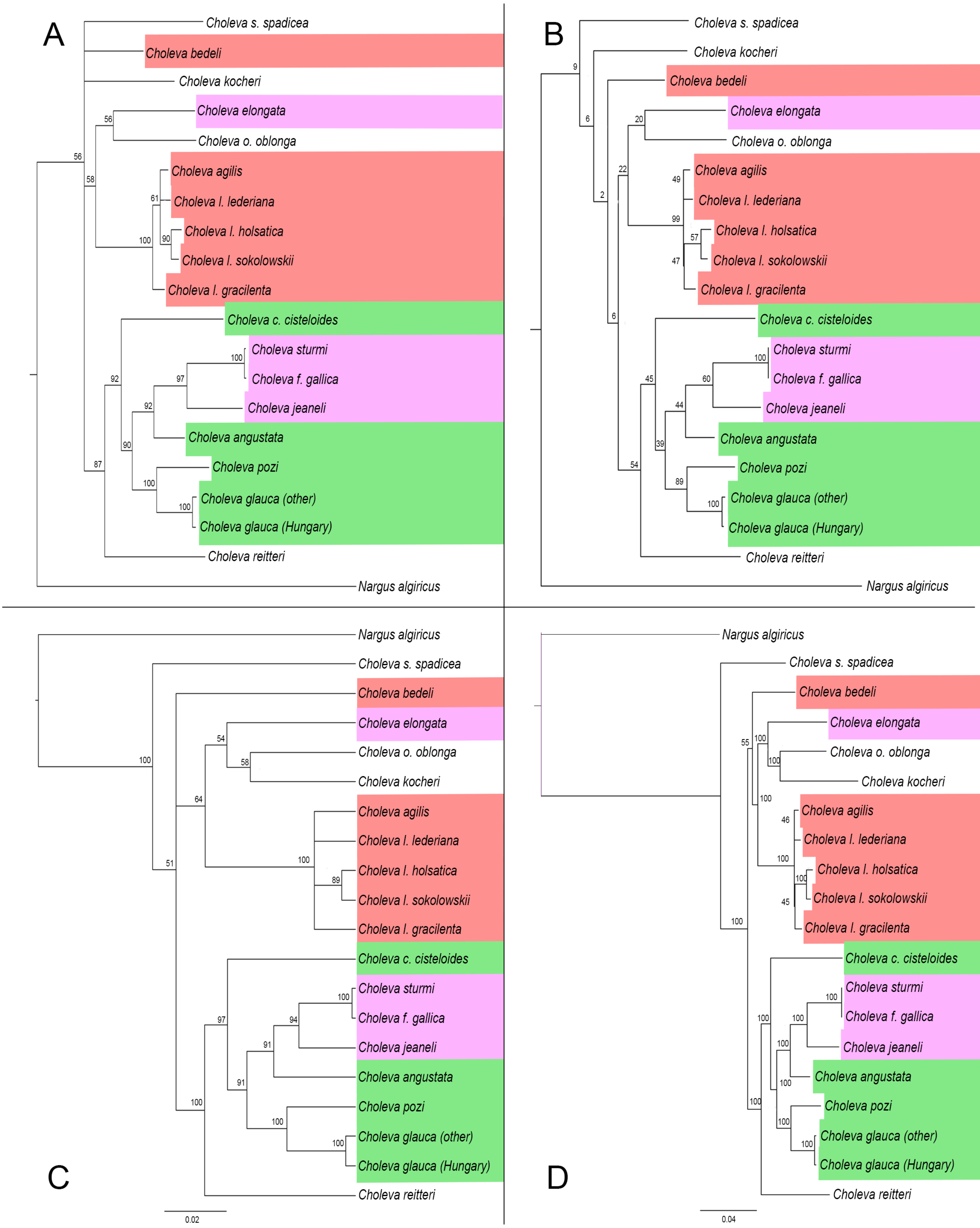


**Supplementary Figure 1**: Phylogenies produced during this study. Figs A and C show a Bayesian tree, whereas B and D are visualizations of maximum-likelihood trees. A clock model was used in C and this tree was used as a constraint in the building of D. Three subgroups within the genus have been marked: *agilis* (red), *sturmi* (pink) and *cisteloides* (green). *Nargus algiricus* was used as an outgroup.
